# Applying the goal programming in the management of the 7P marketing mix model at universities-case study

**DOI:** 10.1371/journal.pone.0260067

**Published:** 2021-11-29

**Authors:** Radoslaw Ryńca, Yasmin Ziaeian

**Affiliations:** Faculty of Management, Departent of Oranization and Management, Wroclaw University of Science and Technology, Wroclaw, Poland; University of Defence in Belgrade, SERBIA

## Abstract

The decision-making relating to effective marketing is less supported by operational methodologies and optimizing methods in high education sectors than in the companies. This paper presents an application of goal programming as an aid for the optimizing marketing mix elements. It describes a project undertaken at one of the Universities in Poland considering the marketing mix includes all 7Ps elements at University. The constraints are first identified based on interviews with academic experts and survey. The analytic hierarchy process method is used to select the forms of promotional activities and Activity-Based Costing method are performed to determine the costs of the activities. Then, based on constraints, the multiple-criteria-programming model is built and applied to construct the marketing mix model at university, and it was solved using LP-Solve software AMPL. A comparison between the newly designed marketing mix and the existing one in terms of each of the criteria shows that the overall objective function could be greatly improved, optimized value can be obtained, and the model can be easily applied in any other high education sectors. The consequence of using the model is the optimal selection (with existing limitations) of promotional activities, taking into account their impact on the perception of the image of the university, significant from the perspective of students and impact on profitability, which is important from the perspective of the university management.

## 1. Introduction

The main goal of marketing at universities and in higher education institutions is to define a quality, market-oriented education system. The marketing strategies and activities of corporate marketing in the education system are much more limited than in other service and manufacturing industries, because of the direct impact one the state regulations defining the education system with established control mechanisms of the accreditation bodies, which prescribe the evaluation system, as well as quality control system. Formulating an institutional marketing strategy for universities involves making decisions about 1. To maintain, build or discontinue the institution’s current programs and markets 2. Future new program and market opportunities. 3. Competitor analysis. 4. Positioning of the institute against competitors. 5. Selecting the target markets and designing the marketing mix [1]. Applying the marketing model is relevant for the universities because it makes possible to improve the university’s competitive position in the market, increase development opportunities or provide services in the direction expected by the university [2]. Therefore, it is necessary to build an effective Marketing Mix model for the universities. The aim of this paper is applying the goal programming in the 7P model at university to improve decision making process the marketing mix.

In another word, high education sectors must anticipate market trends and occupy the right place at the appropriate time, offering opportunities to review new management behaviors, adopting marketing activities that dare to break paradigms [3]. Therefore, for a company or any high education sector is important to establish how it will implement its marketing activities, it must consider the intangible characteristics of the services [4]. So, marketing is seen as a means of obtaining competitive advantage, currently used as strategies by different types of sectors [5]. Taking into account, the 7 P’s of marketing, also known as the marketing mix, were applied to, to achieve strategies related to the market. So, this direct link from the marketing area is the 7 P’s (price, place, product, promotion, process, people, and physical evidence). Through these variables, marketers can establish an effective marketing plan for the organization [6]. To gain these objectives, a proper method should be applied in the evaluation of the criteria. According to purpose of the study, the following research problem is interested: How the management of marketing-mix in high education sectors can be optimized?

As mentioned, having an effective Marketing at Universities requires a special ability to select and access marketing-mix elements. In this research, the goal programming, Analytical Hierarchy Process (AHP) and Activity-based costing method (ABC) have been developed to take into account both qualitative and quantitative factors in multiple sourcing of marketing-mix elements. Goal programming (GP) is an important method in the category of multi-criteria decision models to apply for analyzing and solving problems with conflicting goals. Originally, in the 1950s, Charnes et al. published the popularity, and the application of GP has been raised due to its mathematical simplicity and modeling elegance. In the last few decades, algorithmic developments and computational improvements have contributed significantly to the diverse applications and different variants of GP models [7].

## 2. Literature review and Problem statement

### 2.1. Literature review

#### 2.1.1 Marketing mix in high education institutions

The topic marketing at universities appeared in the mid-1980s as branch of health care marketing [8]. The idea of marketing was accepted, especially after universities had a challenge regarding funding and getting more students [9]. Kotler defined marketing in high education sectors as a process of analyzing, planning, implementation, and control of carefully formulated programs that are intended to bring about a voluntary exchange of values with the target markets and institutional goals. Marketing consists of designing the institute’s offerings to cover the needs and desires of the target market, and applying effective pricing, communication, and sales to inform, motivate, and serve those markets [10]. In 1946 after Neil Borden published the concept of marketing mix in his article, marketing mix theory became very popular in the academic world. The marketing mix is very easy to use and enables marketing to be separated from other company activities and marketing tasks to be delegated to specialists. The components of the marketing mix also have an impact on a company’s competitive position. Hence, it is a powerful concept [11]. The marketing mix has both communicative and operational roles. Communication function is the requirement to convey to users the importance of services to their needs or preferences. The operational function focus on the removing the boundaries in transactions or exchanges so that the users who have selected this service can enter the exchange process with minimum effort [12]. Generally, the 7Ps are as followed: 1) Product, this is the package of benefits that the seller offers, and the customer receives, 2) Price, this is the total cost to the customer of the products, 3) Place, it is the location where the exchange is happening, 4) Promotion, it is the marketing communication package used to make the offer known to potential customers and persuade them to investigate it further, 5) People, who are crucial to success in marketing, particularly in services, where they usually are the product, 6) Process, it is the set of activities that results in delivery of the product benefits, and finally, 7) Physical evidence, it is the lasting proof that the service has happened [13].

According to literature review, following elements of Marketing Mix, 7 P, in high education are listed in Table 1:

**Table 1:** Marketing-mix factors in high education sectors.

Product	Price	Place	People	Process	Promotion	Physical evidence
• Programs for students • Offers for the field of study, postgraduate students, and election courses • Modern and effective teaching methods • Effective schedule regarding expectations of students • High level of teaching • Coordination of teaching content by university • Offers for the specialized courses • The selection of teaching staff appropriate to the content of the curriculum	• Condition of payment • The entry fees at the university • Cost of teaching • Cost of additional extracurricular activities • Access and the amount of scholarship and scholarship system • The amount of government for education at the university • Cost for books and materials • Cost for accommodations	• Location of campus • The attractiveness of the town where the university is located • Access to resources and information • The possibilities of using e-learning • Opportunity to study in long distance • The number of candidates per seat at the university	• Qualifications and achievements of scientific staff • Technical and organizational efficiency of university management • The number and structure of employees in terms of the academic level • Incentive scholarship system • The possibilities of scientific development • Opinion for academic teachers and employees	• The structure of classes • Teaching methods (e.g., Lectures, seminars) • Cooperation with the business environment • International cooperation (student exchanges, lectures visiting) • Cooperation with employees (e.g., Practice) • The possibility of further study at the university • Research projects • Monitoring of careers of graduates	• Public relation activities • Creating the visual identity of the university and favorable relations with the local community • Participation of academics in all kinds of national and international organizations and committees • The attention to external and internal appearance of the university • Publishing own magazine university and handouts • Sponsorship of cultural and sports events, festivals • Using of modern forms of communications (e.g., via the internet)	• Equipment of universities • Infrastructure of library • Access to computer labs • Access to electronic research databases • Equipment of classrooms (e.g., Furniture, audio-visual equipment) • Technical state of scientific and teaching stuff • Center for foreigners • Architecture of the university

Source: Own elaboration based: (R. Ryńca, Z. Malara, Problems of shaping the competitiveness of modern universities. Conditions, instruments, actions. Reports of the Faculty of Computer Science and Management, Wrocław University of Technology. 2015, Ser. PRE-No. 25).

In above table, the most important elements for price, product, promotion place, process, people, and physical evidence are determined in the higher education sector. In different universities, these elements can be defined and described without any limitation [14].

- *Product in high education sectors*. Kotler has specified the curricula and services in the case of educational offers. The provision of services is associated with challenges, since most services are immaterial, inseparable, and adjustable [15]. The nature of the service is important because of its impact on the insight and behavior of the user, and also because it strongly defines the task of other marketing areas, which is necessary for a systematic and successful marketing service [16]. Regarding marketing mix in educational institutions, it is important to determine what are services to offer students, partners, and other citizens. An institutional program and service involve all available programs in the institution. Classes, libraries, computer labs, on-campus lectures are example for educational programs at universities. Sports facilities and clubs, film series, dances could be mentioned as some examples regarding recreational programs. Counseling center and counselors as personal-growth programs and Health center as medical services are different type of services and programs that many universities are offering them [17].
- *Price in high education sectors*. Price is one of the most flexible elements of the marketing mix that has a direct and short-term impact on a company’s profitability and profitability [18]. This component at universities can be defined as an amount of money that the recipient of educational services pays to the institution for the services provided. price included all aspects about tuition fees and other related payments [19].
- *Promotion in high education sectors*. One of the key elements in the marketing mix is promotion. It contains all Marketing efforts to generate public interest in their student’s enrollment at universities. These efforts include the use of social media, websites, face-to-face conversations, and media advertising as a communication link between the high education sectors and the target market [20]. According to Bell (2002) that most high education sectors in the world use publicity (PR) promotions and in this sector such advertisement methods like television and press advertising are not too common [21]. The mechanisms such as PR, Expos and Brochures, as well as some other relevant promotional items, are more useful in public promotion at universities [22].
- *People in high education sectors*. This element refers to all people in the educational sectors who provide services for the students in formal way [23]. The ability, skills, experiences, and knowledge of the academic personnel are some of the most important factors that can be obtained for the satisfaction of students [24]. In another hand, all students who participate in the provision of services, can have impact on a service user’s decisions based on the nature of that service. All employees need to identify the intentions and request of student. The motivation of the employees can be increased through training and further education. An open communication through the institutions is required for maintaining and developing an effective, friendly, and internal communications channel. The research manifested that there is a positive correlation between successful internal communication and the positive attitude of employees in the companies [25].
- *Process in high education sectors*. This component consists of all administrative functions like registration patterns, course, evaluation, examination procedures, result communication and graduations of the university [26]. The teaching-learning processes and the school relaxation policy can be also considered as process at universities.
- *Place in high education sectors*. This marketing mix variable refers to the actual location of the university. Here, the elements such as distance, comfort, and extent are playing an important role. It can also involve the way of academic offer to cover the expectations of students, well as to the virtual access to didactic materials, to the possibility of distance learning [27].
- *Physical evidence in high education sectors*. This component at university involves the totality of the perceptible elements to develop academic activities [28]. Also, it refers to all available university’ physical resources. This physical evidence could reduce the intensity of student service [29]. The facilities of universities and buildings can be considered as accessible physical evidence. A video projector and other equipment required for presenting lectures in classrooms can be obtained as physical evidence [30].

#### 2.1.2 Goal programming

GP is a branch of multi objective optimization, which in turn is a branch of multi-criteria decision analysis (MCDA), also known as multiple-criteria decision making (MCDM). This is an optimization program. It can be thought of as an extension or generalization of linear programming to handle multiple, normally conflicting objective measures. The model enables multiple objectives to be taken into account at the same time, while the decision-making process searches for the best solution from a range of workable solutions [31–35]. Each of these measures is given a goal or target value to be achieved. Unwanted deviations from this set of target values are then minimized in an achievement function. This can be a vector or a weighted sum dependent on the goal programming variant used. As satisfaction of the target is deemed to satisfy the 5-decision maker(s), an underlying satisficing philosophy is assumed. Goal programming is used to perform three types of analysis:

1. Identify the necessary resources to get favorable objectives.
2. Identify the degree of achievement of the goals with the available resources.
3. Providing the best possible solution among a different number of resources and priorities of the goals [36]

#### 2.1.3 Analytic hierarchy process (AHP)

For several years, many techniques have been developed to organize and analyze complex decisions. Multi-Criteria Decision Making (MCDM) is a known methodology in decision making and evaluation. Some of the most famous MCDM tools are as following: AHP, ANP, TOPSIS, ELECTRE, MUSA, AKUTA, VIKOR, PROMETHEE, SAW, COPRAS, SWARA and FARE. A good decision-making model needs to tolerate vagueness or ambiguity because complexities and vagueness are common characteristics in many decision-making problems [37]. Since decision- makers often provide uncertain answers rather than precise values, the transformation of qualitative preferences to point estimates may not be sensible. Conventional AHP that requires the selection of arbitrary values in pairwise comparison may not be sufficient and uncertainty should be considered in some or all pairwise comparison values [38]. Thomas L.Saaty developed AHP in the 1970s and since this time, this method is applied in many studies in decision making for complex situations and scenarios [39,40]. The application of AHP begins with breaking a problem down into a hierarchy of criteria for easier analysis and independent comparison. Once this logical hierarchy has been established, decision makers can systematically evaluate the alternatives by making pairwise comparisons for each of the selected criteria [41]. Flexibility of this method and intuitive appeal to the decision makers are most important advantages of AHP [42], therefore it is conducted in this paper. Pairwise comparison can be found form data input straightforward and convenient, and importance of each criteria will be very clear [43]. Additionally, the bias in decision making process is reducing in this method because of consistency in the mechanism and group decision making is supported by calculating the geometric mean the individual pairwise comparisons [44] Another reason, that this method was applied is its capability to derive scales where no measures normally exist [45]. In another word, this model gives the decision maker room to score each criterion comparing it to other criteria per their own judgement although it subjective. And most researcher prefer AHP because of this feature (scoring criteria) since it is difficult to come by already scored criteria between two or more variable. By reviewing the articles, researchers mainly still use methods such as AHP to obtain the weights of criteria subjectively using pairwise comparisons and it is the most known method for this purpose considering the accuracy and simplicity of calculations. AHP even with its drawbacks, is the best method for the problems like those personal and some corporate, because the needs are supposed to be perfectly known by those who establishes preferences, the DM, and because the consequences of the process fall on those that expressed their wishes and needs.

There are many publications on fuzzy multi-criteria scoring in the literature on this subject. [46–50]. The method enables the evaluation / selection of decision variants on the basis of linguistic variables that are modeled as fuzzy numbers. The respondents can also indicate weightings for individual criteria as well as the characteristics of their preferences and dispositions, which are also modeled using fuzzy numbers [51]. The fuzzy approach makes it possible to better grasp the uncertainty and difficulties than the traditional approach. Since many decisions have to be made in any high education sector based on ambiguous and inaccurate data and preferences, the use of fuzzy numbers can be a helpful tool for managers at universities and colleges. As mentioned in the article, the authors opted for the traditional AHP (not fuzzy) approach because of its simplicity, also being aware of its limitations. As mentioned, the simplicity of this method (AHP) is the main reason for the choice and, on the other hand, that the uncomplicated model and methods have a great chance of success in complex environments such as universities, and this is an advantage of this method. Accessible literature reviews determined that there is no unambiguous classification of the methods of determining the criteria weights [52]. As mentioned, one of the most frequently used methods based on pairwise comparisons is the Analytic Hierarchy Process (AHP) method [53]. The methods of pairwise comparisons, in addition to the AHP method, also include the DEMATEL method [54] and the Best Worst Method (BWM, the resistance to change method [55], which has the elements of the swing method and the pairwise comparison methods. Another new subjective model to determine the weight of criteria is the Full Consistency Method (FUCOM), which its algorithm is based on the pairwise comparison of criteria Target. The famous methods from the group of subjective methods for calculating the weighting values of criteria are the AHP method, the DEMATEL method (Decision-making Trial and Evaluation Laboratory), and the SWARA (Step-Wise Weight Assessment Ratio Analysis) Method [56] and the BWM [57]. There is also a new method, the Model of Weight Assessment Based on Levels (LBWA), which was introduced by Žižović & Pamučar in 2019 for the first time [58]. LBWA has been used in several papers to solve various problems [59,60]. The LBWA method has advantages, that calculation of weighting coefficients can be done with a small number of comparison criteria, it’s a simple algorithm with easy mathematical set-up to obtain the weighting coefficients [61].

- *Applying the AHP method at the university*. Recently, the interest in the application of the analytic hierarchy process (AHP) is increasing in the educational sector and many researchers are focusing on this area [62–69]. The appraisal of a university can be made on the basis of various criteria and by different decision makers. Decision-makers may be guided by completely different selection criteria in their assessment of satisfaction with the university. Due to the fact that the actions taken by the management should be based on reliable and accurate information, it seems justified to use the multi-criteria decision-making method. The AHP method allows for solving decision problems in the discussed cases” [70–73]. As indicated by R. Ryńca and D. Kuchta, the use of the AHP method in a university, whose task is to support decisions in the case of various options, could be a helpful tool in the hands of the university management. It would make it possible to select those forms of promotional activities which are particularly important from the perspective of the evaluators and which should be used in the first place.

#### 2.1.4. The activity-based costing

In the literature on the subject, the method of costing processes and activities is known—activity-based costing (ABC calculation). It allows to estimate the costs of processes and activities, and consequently the costs of cost objects (customer, distribution channel, product). The idea behind ABC assumes that the cause of costs is not resource consumption, but activities that consume resources. Settlement of resources for activities is based on the resource cost drivers. At a later stage, the costs of activities are assigned to cost objects, depending on the degree of "consumption" of activities [74].

The use of activity-based costing is very wide. As R.Kaplan and R.Cooper point out, the information generated by the ABC calculation can be used to make effective decisions. Proper management of activities based on, inter alia, cost information, may consequently contribute to increasing the efficiency of activities and reducing their costs [75]. According to R. Kaplan and R. Cooper, the information obtained thanks to the ABC calculation allows, therefore, to redesign the processes taking place in the enterprise in order to improve their efficiency [76]. According to M. Marcinkowska, activity cost management allows us to understand the processes taking place in the enterprise and the relations between them. It also indicates necessary actions as well as those that can be eliminated [77]. Activity costing can also be used in a university. Since cost management in most universities comes down to basic financial reporting and simplified economic analysis, i.e., a comparison of costs incurred with planned costs, it seems extremely important to use the ABC account to manage the costs of activities and processes in a university. Proper management of the costs of activities would allow for more efficient management of the university, increase the quality of services, and optimize costs. According to the authors of the article, the ABC calculation can also be used to calculate activities in the proposed model. It can be helpful for the calculation of promotional activities [78].

- *The activity-based costing at universities*. Activity-based costing can also apply to an institution of higher education. Its applications can be found in countries such as Great Britain, the United States and Australia [79–81]. In the national literature on the subject, there are few publications on the ABC account in Polish universities. Nevertheless, it can be noticed that the interest in the discussed subject in Poland is growing every year [82]. The literature on the subject knows the publications of the author of the monograph in the field of cost accounting for the university. There are also known studies whose purpose was to determine the state of cost management and effectiveness of Polish universities [83,84]. The analysis of the literature related to the functioning of the university indicates three fundamental processes of the university, they are: Teaching process, Research process, -Professional services process. In addition to the above-mentioned processes, Cox K. points activities related to professional development [85]. The conducted analysis from previous studies allowed to define some activities, which should be taken into consideration during defining the processes carried out by faculty staff [86]. Additionally, one-by-one interviews in marketing department have been conducted to define necessary activities to make promotions. In the Table 2, some examples of activities at universities according to elements of marketing mix, process and promotion was shown.

**Table 2:** Process- and promotion example activities at universities.

Marketing mix element	Activity
**Process**	• Updating and improving knowledge and competence of a faculty member • Participation in specialized training courses • Formulation of a new research problem • Actions to support the teaching process • Preparing lectures • Developing training programs and updating of educational content • Enriching personal knowledge • Professional services • Development of scientific reviews, articles, doctorate, habilitation monograph • Actions with an administrative and supportive character • Presentation and dissemination of results
**Promotion**	• Designing and printing flyers • Preparing content & images for posts on social media (e.g., Tweeter, Facebook, LinkedIn, Instagram) • Creating videos and stories for social media • Free College Advertising Consultation • Scheduling programs on TV or Radio • Training student ambassadors for advertisement on TV and Radio

Source: Own elaboration.

### 2.2 Problem statement

Higher education institutions are increasingly realizing that the university is a service industry and regarding the expectations and needs of their students, they must apply Marketing Mix (7P). It is important to increase student satisfaction because colleges and universities are expanding rapidly, also education fees are increasing [87] and the education market became very competitive. Therefore, universities must adapt their strategy in order to their different offers in comparison with the competitors. In general, according to the unique characteristics of services in high education sectors, marketing is relatively challenging and risky, because evaluating a good service before purchasing for customers is difficult [88]. In such a situation, high education sectors are playing an important role to decrease this risk for their customer and encourage them to make a decision with more confidence [89]. Already, many authors, recognized how important is marketing in student recruitment [90–92], but it is necessary to place a greater emphasis applying an effective marketing model and optimizing the relevant activities.

Based on problem statement, the relevant research question is, how high education sector can optimize their marketing-mix activities to get an effective result in a very competitive market. Purpose of this research is introducing a model by using goal programming to optimize marketing mix elements.

### 2.3 Research gap

The analysis of the publications in databases in the field of the Goal Programming in high education sectors reveals some research gaps and challenges that may occur during the introduction of this topic. In the field of marketing, there are already some papers which are using goal programming [93–98] but the research gaps show that this optimizing model was not applied specifically for the marketing mix at universities. According to this model, there is some research about Academic Plans Design, Resource planning at universities and Strategy of University image [99–106]. However, this study is focusing on this gap for optimizing the Marketing activities by using of the Goal programming.

## 3. Methodology

**Fig 1:**
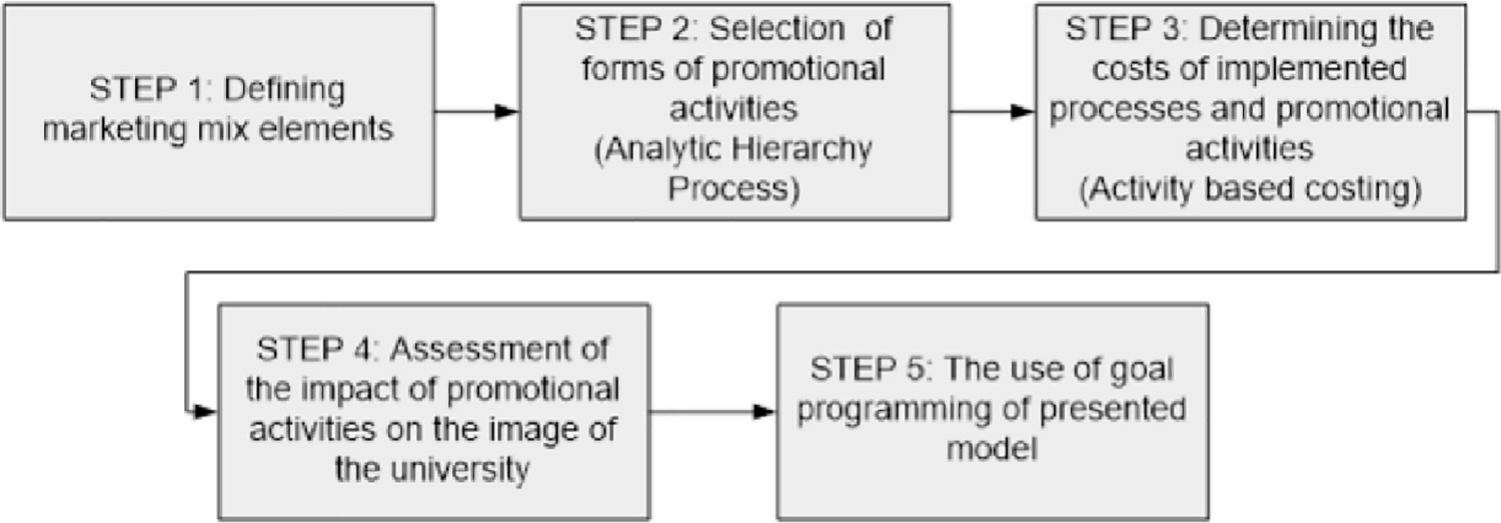
Proposed implementation of the proposed model. Source: Own study.

Fig 1 shows the implementation of the proposed model.

The starting point (**step 1**) should be to define the elements of the marketing mix. During this stage, the products of further analysis should be selected. The product is usually a teaching service provided, a field of study. It is also important to determine the price the service provided, taking into account the profitability of universities and competition on the educational services market. It is also important to determine the internal capabilities of the university in terms of the number of students in a given field of study or the method of providing the service (full-time, part-time studies).

**Step 2** is the selection of forms of promotional activities in which the university plans to conduct promotional activities. This stage may be a problem for the university management, taking into account the complexity of the forms and their different characteristics (e.g., the scope of the form of promotion or its cost). In this case, the use of hierarchical process analysis (AHP) can be helpful.

**Step 3** is the determination of the costs of the teaching service provided, in particular of the teaching process carried out at the faculty. It is also important to draw a budget for promotional activities and the calculation of promotional activities as part of the marketing mix. The use of activity-based costing (ABC) may be helpful in this respect.

**Step 4** is the assessment of the impact of the promotional activities carried out on the image of the university. This stage should be carried out by conducting a survey on a group of students of a given field of study. The aim of the study is to determine the impact of promotional activities on the image of the university.

**Step 5**. The use of targeted programming. This stage consists in formulating the objective function and the limitations of the proposed model.

According to the authors of the article, the presented model above may bring many **benefits** and be very helpful. It provides information on what costs the university has to incur to ensure the implementation of a certain number of promotional activities with the existing financial constraints. Thanks to the use of goal programming in university management, it is possible to provide the management staff with information on the necessary number of students in order to ensure the profitability of the university at the planned level. It allows for the optimal selection of such promotional activities that will ensure the assumed image level of the university from their implementation, which is important for e.g., students. The proposed model is also open-ended. This means that the university management can take into account a number of different variables and limitations depending on its own decision-making needs.

The authors are also aware of the **weaknesses**. The application of the proposed model requires knowledge of the costs of teaching process i.e., the preparation of the ABC calculation. As mentioned before, this can cause a number of difficulties and limitations. The first is the high cost of developing a calculation using the ABC account. Difficulties with access to cost data may be of particular importance. The "human factor" may also be of importance, as it may become a serious barrier preventing the application of the cost accounting method in a university. Therefore, in the case of the human factor, the great support, commitment and determination of the university management in the work on the ABC account may be crucial [107]. It requires the selection of forms of promotional activities, taking into account the AHP method. Among other things, this may cause difficulties with the knowledge of the calculation procedure, which may not be understandable for all managers. When there are a large number of variables, appropriate software is recommended, which can be costly.

In order to use the proposed model, it is also necessary to know the assessment of the impact of promotional activities on the evaluation of the image of the university from the perspective of students. This requires survey research, analysis, and subsequent periodic update, which in turn may result in high costs related to the implementation of the model and be time-consuming.

### 3.1 Goal programming in marketing mix at universities

Proper management of a university requires the use of methods and tools that support making effective sustainability decisions. This article proposes the use of purposeful programming to solve a decision problem in the 7P model that may be encountered by university management. In Table 3, the marketing mix model in the university is presented. Selected information that may be relevant for the management in the university management process:

**Table 3:** Marketing mix model based on goal programming at universities.

Product	Field of study 1	Field of study 2	Field of study 3	…	Field of study *i*	The assumed level of the achieved goal
**Price**	Tuition fee *C*_1_ in *K*_1_ field of study	Tuition fee *C*_2_ in *K*_2_ field of study	Tuition fee *C*_3_ in *K*_3_ field of study	…	Tuition fee in *K*_*i*_ field of study	*-*
**Promotion**	Costs of *p*_*j*1_ promotional activity in the *K*_1_ field of study	Costs of *p*_*j*2_ promotional activity in the *K*_2_ field of study	Costs of *p*_*j*3_ promotional activity in the *K*_3_ field of study	…	Costs of *p*_*ji*_ promotional activity in the *K*_*i*_ field of study	*M*
**Place**	Number of forms of education *f*_1_ in *K*_1_ field of study	Number of forms of education *f*_2_ in *K*_2_ field of study	Number of forms of education *f*_3_ in *K*_3_ field of study	…	Number of forms of education *f*_*i*_ in *K*_*i*_ field of study	*-*
**People**	Number of students *l*_1_ in *K*_1_ field of study	Number of students *l*_2_ in *K*_2_ field of study	Number of students *l*_3_ in *K*_3_ field of study	…	Number of students *l*_*i*_ in *K*_*i*_ field of study	*P*
**Process**	Costs of the didactics process *kpk*_1_ in *K*_1_ field of study	Costs of the didactics process *kpk*_2_ in *K*_2_ field of study	Costs of the didactics process *kpk*_3_ in *K*_3_ field of study	…	Costs of the didactics process *kpk*_*i*_ in *K*_*i*_ field of study	*-*
**Physical evidence**	The level of assessment of the image of *O*_*j*1_ from the implementation of promotional activity in the *K*_1_ field of study	The level of assessment of the image of *O*_*j*2_ from the implementation of promotional activity in the *K*_2_ field of study	The level of assessment of the image of *O*_*j*3_ from the implementation of promotional activity in the *K*_3_ field of study	…	The level of assessment of the image of *O*_*ji*_ from the implementation of promotional activity in the *K*_*i*_ field of study	*O*

Source: Own elaboration.

Table 3 presents proposals for components of the 7P model at the University. In terms of the product aspect, the university provides a didactic service on and in these fields of study i. The price of the service is expressed in the level of tuition fees at the i-th course. By distribution is meant the mode/form of service provision. The university can provide classes in full-time, part-time, remote or hybrid mode (due to e.g., covid-19). In terms of the human factor, the proposed model considers the clients of the provided didactic service—students. Not without significance are the processes implemented at the university, including the educational process, and supporting processes. To maintain the planned level of profitability, the costs calculations based on the activity costing method may be important. The management of the university should also be aware of the material evidence of the perception of *O*_*j*_’s image, resulting from the marketing activities in the i-th field of study.

Undertaking marketing activities, including promotional activities in the i-th field of study, relates to bearing the cost of the j-th activity. Due to the financial limitations of the university, the implementation of all promotional activities may be difficult. Therefore, it is necessary to choose such activities that would allow to maintain the expected level of R profitability of the university with the assumed level of the budget for marketing activities M.

The aspect that has an impact on the university’s source of income also seems to be important. In particular, the information on subsidies from the state budget, per student in the i-th major, or the number of students in the i-th major, may prove important. The managers of the university should therefore strive for a situation in which it is possible to increase the number of students regarding the number of available places at the university P. The price of the service provided is also important, i.e., the level of tuition fees at the i-th faculty as well as the number of modes/forms of education fi. Therefore, it is right for the university management to make decisions in such a way that they take into account the existing limitations in which the university operates with a view to achieving its goals in an optimal way.

The goal function of the decision-making model is to minimize the weighted sum of unfavorable deviations from the established levels of goal achievement:

$$y_{1}^{+}+y_{2}^{+}+y_{3}^{-}+y_{4}^{-}\to min$$ (1)

Therefore, the management of the university should focus its activities on maintaining the costs of marketing activities at a level not higher than M.

The first limiting condition of the proposed model is in its form:

$$\sum_{ij=1}^{m}d_{ji}*k_{ji}-y_{1}^{+}+y_{1}^{-}=M$$ (2)

where:

*d*_*ji*_-binary decision variable, related to the implementation of the i-th marketing activity in the i-th direction

*k*_*ji*_-a constant that expresses the cost of the j-th marketing activity- in the i-th field of study

$y_{1}^{-}$-the amount by which the achieved cost level is less than *M*

$y_{1}^{+}$-the amount by which the achieved cost level exceeds *M*

*M*-the size of the budget allocated for the execution of image-building activities

It is also important to plan the number of places on the faculties, bearing in mind the limited number of P. The limited number of places may result, in the case of public universities, from the availability of teaching staff. In the case of non-public universities, for example, due to infrastructure or human resources limitations, as well as the adopted strategy of university development.

The second limiting condition of the proposed model:

$$\sum_{i=1}^{m}l_{i}-y_{2}^{+}+y_{2}^{-}=P$$ (3)

where:

*l*_*i*_-the decision variable of the number of students in the i-th field of study

$y_{2}^{-}$-the size by which the number of students is less than P

$y_{2}^{+}$=the size by which the number of students is higher than P

*P*-level of available places on the directions.

It is extremely important in the university management process to maintain the profitability of the university at R level.

The third limiting condition of the model takes the form:

$$(\sum_{i=1}^{m}\left(c_{i}*f_{i}*l_{i}\right))-(\sum_{ji=1}^{m}d_{ji}*k_{ji}+\sum_{i=1}^{m}{k_{p}k}_{i})-y_{3}^{+}+y_{3}^{-}=R$$ (4)

where:

*c*_*i*_-a fixed rate informing about the amount of tuition fees in the i-th direction

*f*_*i*_-constant informing about the number of forms of education in the i -th field of study

*l*_*i*_-decisional variables of the number of students in the i-th field of study

*d*_*ji*_-a permanent information about the level of the subsidy from the state budget (per student) for the i-th major.

*k*_*ji*_-constant informing about the level of the cost of j-th- marketing activity on the i-th field of study

*kpk*_i_-constant informing about the level of costs of processes carried out in the i-th field of study

$y_{3}^{-}$-the amount by which the university’s profitability level is less than *R*

$y_{3}^{+}$-the amount by which the university’s profitability level is higher than *R*

*R*-constant informing about the assumed level of university profitability.

The selection of marketing activities should also consider the assessment of the image of the university from the perspective of students and candidates for studies resulting from the conduct of this activity in the i-th field of study.

The implementation of marketing activities should allow to maintain the assumed level of evaluation of the image of the university.

The fourth limiting condition of the model assumes the form:

$$\sum_{ji=1}^{m}d_{ji}*o_{ji}-y_{4}^{+}+y_{4}^{-}=O$$ (5)

where:

*d*_*ji*_—binary decision variable, related to the implementation of the i-th marketing activity in the i-th direction

*o*_*ji*_—constant informing about evaluation of the i-th field of study from the perspective of students and candidates for studies from the implementation of j-th marketing activity

*O*—constant informing about evaluation of the faculty’s image from the perspective of students and candidates for studies from the introduction of all marketing activities

$y_{4}^{+}$—the amount by which the level of assessment of the faculty’s image from the perspective of students and candidates for studies from the introduction of marketing activities is higher than the *O*

$y_{4}^{-}$—the amount by which the level of assessment of the faculty’s image from the perspective of students and candidates for studies from the introduction of marketing activities is lower than *O*

The proposed decision-making model is not a closed one. It may also apply to image choices or improvement actions in the university. The thesis that the management of the university should consider as many factors and variables as possible in the decision-making process seems to be correct. This will enable full and sustainable management with existing limitations resulting from both the internal potential of the university and its environment. It seems that using targeted programming in this case would be helpful.

## 4. Case study

The model below was developed at university in the Dolnośląskie province in Poland. "In the conducted study, the consent of the unit participating in the study was not needed. The study was not conducted on minors. There was no research ethics committee at the time of the study in the analyzed organization. The management was informed about the survey and gave its verbal consent. One of the authors of the article was the Vice-Dean of the Faculty at that time”

The starting point for the implementation of the proposed model was the definition of products within the marketing mix (**step 1** in Fig 1). A product at a university is the teaching service provided. At this university, the teaching service is provided in three fields of study: management, law, and tourism and recreation (Table 4). The tuition fees for individual fields of study are between PLN 4,200 and 5,500. The internal capabilities of universities were also determined in terms of the number of students in given fields of study or the method of providing the service (full-time, part-time studies). The limit of new students in the faculties is 1,200 in total. The next step (**step 2**) consisted in selecting the forms of promotional activities in which the university plans to conduct promotional activities.

**Table 4:** Matrix of pairwise comparisons for the criterion "cost of the form of promotion".

Matrix of pairwise comparisons					Normalized matrix of pairwise comparisons				Preference indexes
	Social Media	Flyers	Radio	Bilboards	Social Media	Flyers	Radio	Bilboards	
Social Media	1,00	3,00	4,00	6,00	0,571	0,667	0,429	0,429	52,38%
Flyers	0,33	1,00	4,00	4,00	0,190	0,222	0,429	0,286	28,17%
Radio	0,25	0,25	1,00	3,00	0,143	0,056	0,107	0,214	13,00%
Billboards	0,17	0,25	0,33	1,00	0,095	0,056	0,036	0,071	6,45%
SUM	1,75	4,50	9,33	14,00	1,000	1,000	1,000	1,000	100,0%

Source: Own elaboration.

**Fig 2:**
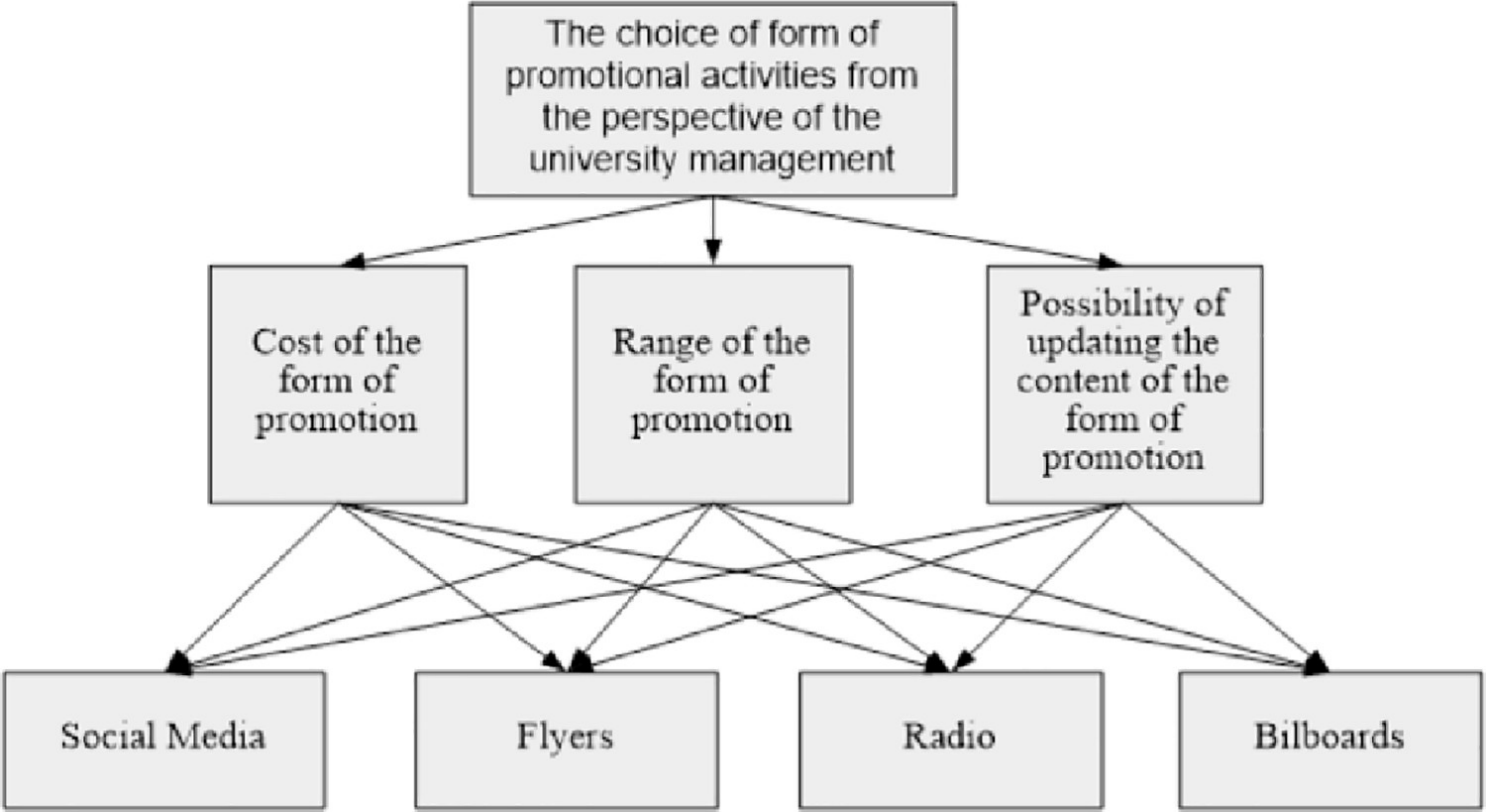
Scheme of assessing the importance of individual forms of promotional activities. Source: Own study.

The starting point for the use of the proposed model was the selection of forms of university promotion activities, which should be given special attention. In the case at hand, the university management took into account the following forms of promotional activities: promotional activities in social media, flyers, press advertising (radio) and billboards. The AHP method was used to determine the weights of individual forms of promotional activities. Fig 2 shows the problem in the form of a hierarchical structure.

Three criteria were indicated, by means of which the proposed solutions will be assessed, such as the cost of the form of promotion, the range of the form of promotion, the possibility of updating the content of the form of promotion. The second step of the AHP method was to perform assessments within a hierarchical structure. These assessments were made by making pairwise comparisons at each level of the hierarchical structure. For the purposes of this step, a structured interview with the university management was conducted. The purpose of the interview was to provide information on the forms of promotional activities that are of particular importance and should be taken into account in the proposed model. The forms of promotional activities were compared in pairs due to each criterion using the 1–9 scale. Where 1- no advantage of one variant over the other, 9-extreme advantage of one variant over the other. A standardization process was also carried out.

As shown in Table 4, the most important form of promotion, taking into account the cost criterion, is social media. Similar calculations are presented for the remaining criteria (Tables 5 and 6).

**Table 5:** Matrix of pairwise comparisons for the criterion "range of the form of promotion".

Matrix of pairwise comparisons					Normalized matrix of pairwise comparisons				
	Social Media	Flyers	Radio	Bilboards	Social Media	Flyers	Radio	Bilboards	Preference indexes
Social Media	1,00	1,00	8,00	5,00	0,430	0,400	0,640	0,357	45,68%
Flyers	1,00	1,00	3,00	6,00	0,430	0,400	0,240	0,429	37,47%
Radio	0,13	0,33	1,00	2,00	0,054	0,133	0,080	0,143	10,25%
Billboards	0,20	0,17	0,50	1,00	0,086	0,067	0,040	0,071	6,60%
SUM	2,33	2,50	12,50	14,00	1,000	1,000	1,000	1,000	100,0%

Source: Own elaboration.

**Table 6:** Matrix of pairwise comparisons for the criterion "possibility of updating the content of the form of promotion".

Matrix of pairwise comparisons					Normalized matrix of pairwise comparisons				
	Social Media	Flyers	Radio	Bilboards	Social Media	Flyers	Radio	Bilboards	Preference indexes
Social Media	1,00	8,00	6,00	9,00	0,713	0,800	0,643	0,600	68,89%
Flyers	0,13	1,00	2,00	2,00	0,089	0,100	0,214	0,133	13,42%
Radio	0,17	0,50	1,00	3,00	0,119	0,050	0,107	0,200	11,90%
Billboards	0,11	0,50	0,33	1,00	0,079	0,050	0,036	0,067	5,79%
SUM	1,40	10,00	9,33	15,00	1,000	1,000	1,000	1,000	100,0%

Source: Own elaboration.

When analyzing the criterion of the scope of the form of promotion, social media and leaflets turned out to be the most important for the management. On the other hand, the billboards are of little importance. Similar calculations were made for the criterion of the possibility of updating the content of the form of promotion. The results are presented in Table 6.

The criteria were assessed in a similar way. Normalization consisted in determining the share of a given criterion in the total calculated by adding values in the columns for each of the variants. The developed matrix of pairwise comparisons, normalized matrix and individual ranking of the criteria are presented in Table 7.

**Table 7:** Matrix of pairwise comparisons, normalized matrix and individual ranking of criteria.

**Pairwise comparison matrix for evaluation criteria**			
	The cost of the form of promotion	The range of the form of promotion	Possibility to update the content of the promotion form	
The cost of the form of promotion	1,00	3,00	4,00	
The range of the form of promotion	0,33	1,00	4,00	
Possibility to update the content of the promotion form	0,25	0,25	1,00	
SUM	1,58	4,25	9,00	
**Normalized matrix of pairwise comparisons for evaluation criteria**				**Preference indexes**
The cost of the form of promotion	0,632	0,706	0,444	59,40%
The range of the form of promotion	0,211	0,235	0,444	29,68%
Possibility to update the content of the promotion form	0,158	0,059	0,111	10,93%

Source: Own elaboration.

The next step was to prepare a ranking of the forms of promotional activities (Table 8).

**Table 8:** Multi-criteria ranking of areas.

Rank of form of promotion	Promotion form / Criterion	Preference indexes
1	Social Media	52,20%
3	Flyers	29,32%
2	Radio	35,15%
4	Billboards	6,42%

Source: Own study.

As a result of the analysis, it was found that the most important form of promotional activities from the point of view of the analyzed criteria are social media. Detailed calculations are presented only for social media:59,40%*52,38%+29,68%*45,68%+10,93%*68,89%. For the next stage, it was decided to take the three most important forms of promotional activities, such as social media, flyers and radio.

The consistency of the evaluation matrix of comparisons with pairs of criteria was also checked. In all cases, the cohesion coefficient did not exceed 10%.

**In step 3**, the costs of the teaching service provided were determined, in particular the teaching process implemented at the faculty. Activity-based costing was used for this purpose.

The starting point for the development of the calculation was the identification of processes and activities in the area of education. In the literature on the subject, there are many examples of the development of identified processes. For the purposes of this calculation, the suggestions of the dictionary of actions by A. Klaus-Rosińska and M. Kowalski were used [108,109]. Table 9 shows the process and its activities in the field of education:

**Table 9:** Resources and activities included in the education process.

Resources	Activities	% of total costs
Professor’s salaries Assistant professor’s salaries (Adjunct) Assistant’s salaries	TOTAL	100%
	I. Conducting classes	50%
	II. Activities directly related to the didactics	40%
	1. Designing classes	15%
	2. Ongoing preparation for classes	10%
	3. Ongoing monitoring of students’ learning progress	5%
	4. Preparation of exams / tests	3%
	5. Completing exams / final tests / assignments	7%
	III. Activities of teaching process not directly connected with courses	10%
	1. Consulting students	6%
	2. Supervising graduate students	3%
	3. Consulting/taking part in scientific associations and student organizations	1%

Source: Own elaboration.

Based on the interview with the university management, the costs of the education process were also calculated based on the resource cost carrier, which was the working time of a research and teaching employee.

In addition to the activity—Conducting didactic classes, which includes conducting lectures, exercises and laboratories, activities related directly to the didactic classes were distinguished, such as designing didactic classes consisting, among others, in the development of a course card, syllabus, preparation of updating materials, preparation of a multimedia presentation. Table 9 also presents activities related to students, such as, for example, ongoing monitoring of students ’progress in learning, checking students’ work, discussing the results of work, and activities related to assessing and crediting the course, which include the preparation and checking of exams / final tests.

The resource cost pools that consume the activities have also been identified. The amount of resource costs was calculated based on the following algorithm:

$${PK}_{z}=\{M*[L_{p}*W_{p}+L_{ad}*W_{ad}+L_{s}*W_{s}]\}*F$$ (6)

Where:

*PK*_*z*_—total pool of resource costs

*M*—the number of months connected to didactic work in the semester

*L*_*p*_—number of professors at the university

*L*_*ad*_—number of assistant professors (adjunct)

*L*_*s*_—number of assistants

*W*_*p*_—the average monthly salary of a professor

*W*_*ad*_—average monthly salary of an assistant professor (adjunct)

*W*_*s*_—average monthly salary of an assistant.

*F*—percentage of salary costs directly related to process of teaching.

In the discussed case, it was assumed that percentage of salary costs directly related to process of teaching constitutes 60%. (60% of an employee’s working time is spent in teaching and 40% in research). The authors are aware of the simplified model of the presented calculation. Bearing in mind that in the analyzed case the costs of personnel remuneration constitute approximately 80% of all university costs. The remaining 20% costs are support and infrastructure costs. The presented ABC model does not take into account the costs of didactic infrastructure used for didactic purposes, i.e., lecture rooms, computer labs or specialized laboratories. Also, indirect costs related to, lecture halls, university administration, which should be taken into account in the case of precise ABC calculation, are not included. The aim of step 3 of the presented model was not to precisely define the cost components of the process of activities, only the total cost of the education process, which in this case study is only of a demonstrative nature. The cost data of the education process for individual fields of study are shown in Table 12. As it shown, it is 230,000 for the field of management, -180,000 for the field of tourism and recreation, and 330,000 PLN for the field of law in business.

In step 3, the costs of promotional activities were also determined (Table 10).

**Table 10:** Costs of promotional activities.

Costs of promotional activities in the field of study: Management	Costs of promotional activities in the field of study: Tourism and Recreation	Costs of promotional activities in the field of study: Business law
*A*_11_: 13.000 PLN	*A*_12_: 15.000 PLN	*A*_13_: 2.000 PLN
*A*_21_: 7.000 PLN	*A*_22_: 10.000 PLN	*A*_23_: 2.500 PLN
*A*_31_: 3.000 PLN	*A*_32_: 6.000 PLN	*A*_33_: 1.500 PLN.

Source: Own elaboration.

**Step 4** consisted in assessing the impact of promotional activities on the image of the university.

For this purpose, a questionnaire survey was conducted on a group of 30 students in three fields of study. The aim of the study was to determine the impact of promotional activities on the image of the university. The respondents rated the impact of a given activity on a scale of 1 to 5, where 1- low impact, 5-high impact. An example of survey questionnaire is presented below (Table 11).

**Table 11:** Questionnaire for the survey in the field study of management.

Indicate the impact of the following promotional activity on the image of a university on a scale of 1 to 5, where 1- low impact, 5-high impact	1	2	3	4	5
*A*_11_: Radio media advertising activities					
*A*_21_: Activities in social media					
*A*_31_: Flyers					

Source: Own study.

The averaged results of the impact of the three promotional activities on the image of the university are shown in Table 12.

**Table 12:** Marketing mix model based on goal programming at universities in selected university.

Product	Management	Tourism and Recriation	Business law	The assumed level of the achieved goal
**Price**	5550 PLN	4200 PLN	5400 PLN	*-*
**Promotion**	*A*_11_:Radio media advertising activities	*A*_12_:Radio media advertising activities	*A*_13_:Radio media advertising activities	*75000 PLN*
	*A*_21_:Activities in social media	*A*_22_:Activities in social media	*A*_23_:Activities in social media	
	*A*_31_:Flyers	*A*_32_:Flyers	*A*_33_:Flyers	
**Place**	Part time studies	Part time studies	Part time studies	*-*
**People**	400 students	500 students	300 students	*1200* students
**Process (cost)**	230000 PLN	180000 PLN	330000 PLN	*-*
**Physical evidence**	The level of assessment of *A*_11_:3.25 The level of assessment of *A*_21_:4.7	The level of assessment of *A*_12_:3.76 The level of assessment of *A*_22_:4.56	The level of assessment of *A*_13_:3.88 The level of assessment of *A*_23_:4.2	*The assumed level of satisfaction (88%)*

*A_*ji*_: j-th marketing activity- in the i-th field of study.

Source: Own elaboration.

As shown in Table 12, in the field of management, the activities *A*_21_: Activities in social media and the activity (4.7) and *A*_11_: Radio media advertising activities (3.25) have the greatest impact on the image of the university. In the field of tourism and recreation—*A*_22_: Activities in social media (4.56) and *A*_12_: Radio media advertising activities (3.76), while in the field of law in business—*A*_23_: Activities in social media (4.2) and *A*_13_: Radio media advertising activities (3.88).

**In step 5,** a model was developed in the form of the formulation of the objective function and constraints.

In the Table 12, the fields and values related each field are shown:

The objective function of the proposed model:

$$y_{1}^{+}+y_{2}^{+}+y_{3}^{-}+y_{4}^{-}\to \min$$

The university plans to undertake several promotional activities (only three have been selected for the purposes of this study): advertising activities in the media, social media activities and flyers. The cost of promotional activities in the fields of study is presented in Table 10. The estimated budget for promotional activities is PLN 75,000.→

Regarding above explanation, the presented model is as follows:

13000*d*_11_+15000*d*_12_+2000*d*_13_+7000*d*_21_+10000*d*_22_+2500*d*_23_+3000*d*_31_+6000*d*_32_+1500*d*_33_$-y_{1}^{+}+y_{1}^{-}$ = 75.000 where:

*d*_11_-binary decision variable associated with the execution of the activity *A*_11_

*d*_12_-binary decision variable associated with the execution of the activity *A*_12_

*d*_13_-binary decision variable associated with the execution of the activity *A*_13_

*d*_21_-binary decision variable associated with the execution of the activity *A*_21_

*d*_22_-binary decision variable associated with the execution of the activity *A*_22_

*d*_23_-binary decision variable associated with the execution of the activity *A*_23_

*d*_31_-binary decision variable associated with the execution of the activity *A*_31_

*d*_32_-binary decision variable associated with the execution of the activity *A*_32_

*d*_33_-binary decision variable associated with the execution of the activity *A*_33_

$y_{1}^{-}$-amount by which the achieved level of costs is lower than 75.000 PLN

$y_{1}^{+}$-amount by which the achieved level of costs exceeds 75.000 PLN

The competences of the number of candidates in given fields of study were also defined. 400 people in management. In the field of tourism and recreation -500 people and in the direction of the right in business—300 people.

Considering the limitations of the number of candidates for individual directions, the model limitation will be as following:

$$l_{1}+l_{2}+l_{3}-y_{2}^{+}+y_{2}^{-}=1200$$

where:

*l*_1_-decisional variables of the number of students in the field 1≤ 400

*l*_2_-decisional variables of the number of students in the field 2 ≤500

*l*_3_-decisional variables of the number of students in the field 3 ≤ 300

$y_{2}^{-}$—the amount by which the achieved level of the number of students is higher than 1200

$y_{2}^{+}$—the amount by which the achieved level of the number of students is lower than 1200

Also, the university assumed the profitability level of PLN 1.200.000 (University revenues from teaching services provided—total costs = 1.200.000 PLN).

5500*l_1_+4200*l_2_+5400*l_3_−[230,000+180,000+330,000] +13000*d*_11_+15000*d*_12_+

2000*d*_13_+7000*d*_21_+10000*d*_22_+2500*d*_23_+3000*d*_31_+6000*d*_32_+1500*d*_33_$-y_{3}^{+}+y_{3}^{-}$ = 1.200.000 where:

$y_{3}^{-}$—the amount by which the achieved level of profit is lower than 1.200.000

$y_{3}^{+}$—the amount by which the achieved level of profit is higher than 1.200.000

*l*_1_≤ 400, *l*_2_≤ 500, *l*_3_≤300

The promotional activities, which are considered in this study, have an impact on the perception of the image by candidates for studies. The management of the university assumed that the assessment of the image should be at the level of 88% complete satisfaction with the implementation of all image-building activities. The proposed restrictions presented as followed:

3.25*d*_11_+4.7*d*_12_+2.3*d*_13_+3.76*d*_21_+4.56*d*_22_+2.7*d*_23_+3.88*d*_31_+4.2*d*_32_+3.1*d*_33_$-y_{4}^{+}+y_{4}^{-}$ = 40 where:

$y_{4}^{-}$—amount by which the level/rating of the university’s image is lower than 88%

$y_{4}^{+}$—amount by which the level/rating of the university’s image is higher than 88%

## 5. Results and discussion

**Fig 3:**
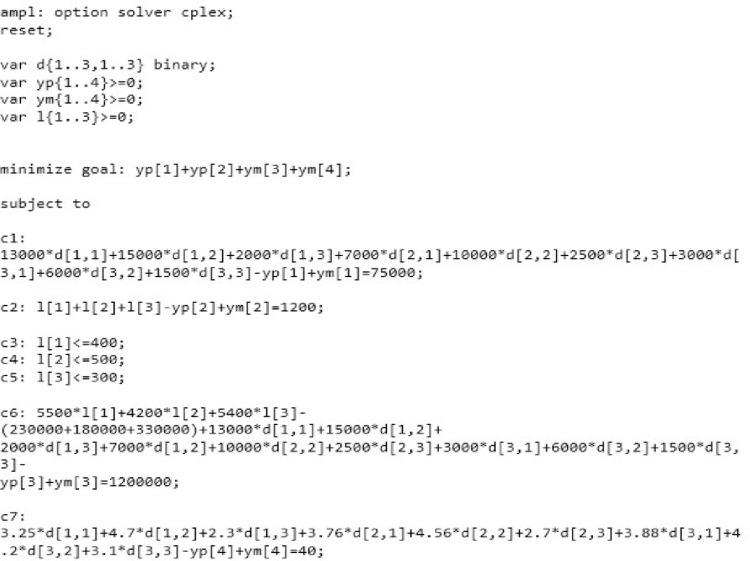
The formulated model in AMPL. Source: Own elaboration.

**Fig 4:**
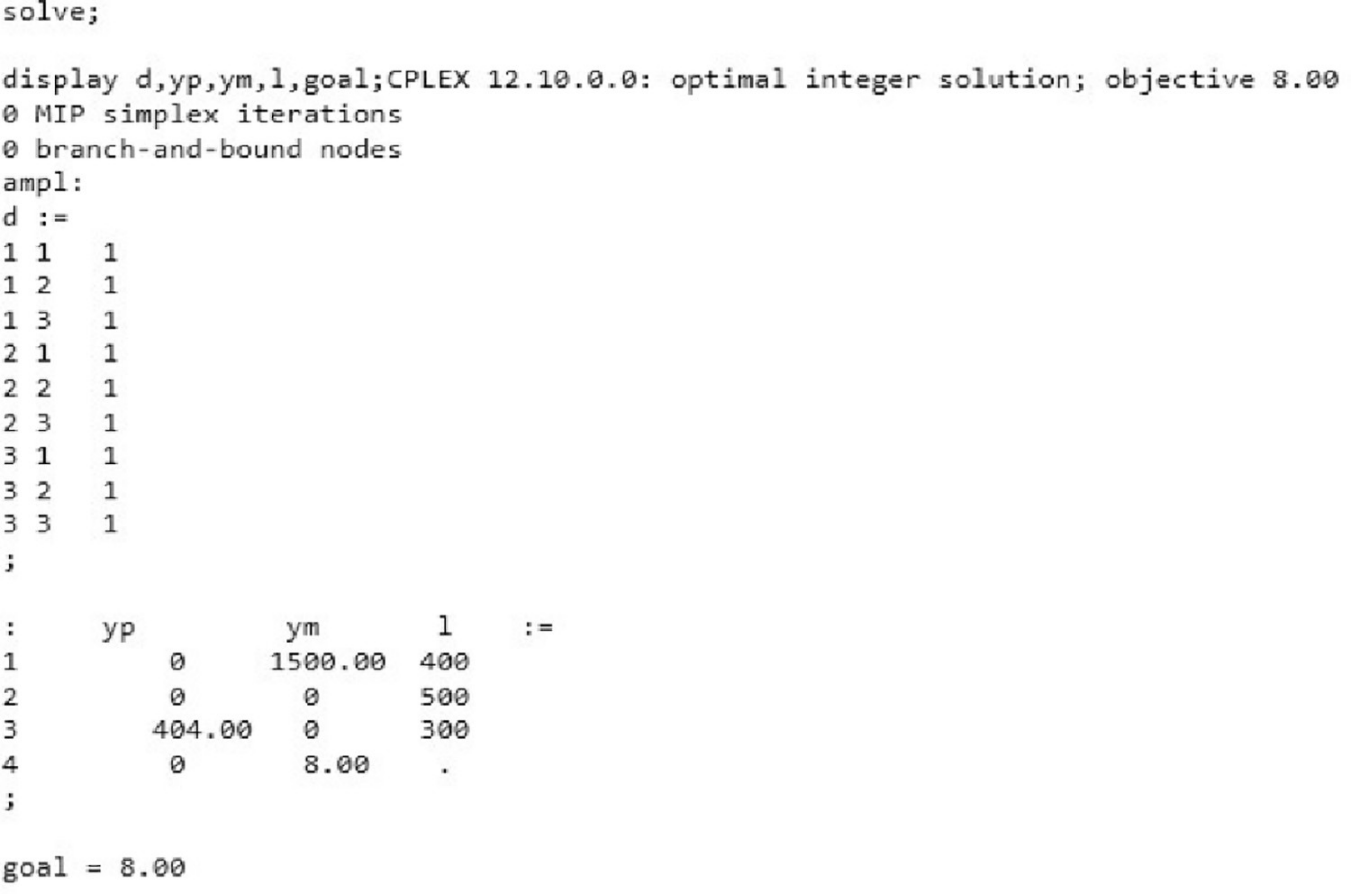
The optimal solution for the objectives assumed in AMPL. Source: Own elaboration.

Linear Programming (LP) is one of the easiest ways to perform optimization and it helps solving very complex optimization problems by making assumptions. It is a method for achieving best outcome with a range of constraints. Which are defined mathematically [110]. For this aim, AMPL is used in this study as the software. AMPL (A Mathematical Programming Language) is an algebraic modeling language for describing and solving highly complex problems for large-scale mathematical calculations [111]. The model on AMPL is as followed in Fig 3:

In Fig 4, the optimal solutions are determined:

The goal programming model is developed from the perspective of the decision-maker at university and goals/targets are set for each of the objectives, and upward deviations are represented with plus (+) superscripts while negative (-) superscripts are used to represent downward deviations. According to the calculation, the budget in the amount of 75.000 (PLN) allows implementing marketing-mix elements. As a result of the execution of these elements, it is possible to have a surplus of 1500 PLN in relation to the budget planned. As, it was important to plan the number of places on the faculties, considering the limited number of 1200 students, the execution of the marketing-mix elements allows acquiring 1192 students in all faculties, which is less by 8 people than the planned number. In order to be able to acquire the planned number of students, it is necessary to increase the budget by PLN 1400 only. The above stated solution indicates that only the number of students needs to be optimized and remained goal for other activities are satisfied. Hence no further optimization is needed.

## 6. Limitation

This study has potential limitations. To apply all mentioned methods, more time and thought is required in the construction of the final model. The authors of the article are also aware of the weaknesses of the use of goal programming. This method can be time consuming to implement. It requires knowledge of the costs of teaching process and promotional activities and access to software in order to make calculations. It also requires skills related to the development of a mathematical model.

## 7. Conclusion

The study shows that in comparison with the current marketing mix elements of the university, the proposed one performs better with respect to the overall objective function and thus achieving a balance between the conflicting objectives. However, there might be some limitations for applying of the model. The advantage of this model is that it allows the marketing manager to discover any important constraints that were not included in the existing marketing model, like what could be the optimal amount to implement the marketing elements. The linear programming model is also flexible and new constraints or goals can be easily added or changed to test the results. The proposed methodology can be very useful for other high education sectors and it is very conceivable that some of the goals and constraints may not be taken into account. The presented proposal of using goal programming can be widely used in a university. It allows for the selection of the optimal solution under the existing assumptions, i.e.. the degree of satisfaction of entities with the selection of promotional activities, profitability, and the number of available places at the faculties. It provides information on what costs the university has to incur to ensure the implementation of a certain number of promotional activities. Provides information about the number of students to ensure a profitability level at a predetermined level. It allows you to select such promotional activities that will ensure the assumed level of satisfaction, which is important for the evaluators. As a summary, it can be mentioned that the novelty of this research is the combination of different methods in the high education sector and create a new model to optimize marketing mix at the university. As further research, this model can be applied for different level of strategic management in higher education sectors. The further stage of the authors’ research will be the development of the proposed model in terms of the impact of improvement activities undertaken at the university on the assessment of various groups of stakeholders (students, employees). Increasing the variety of proposed promotional activities and assessing their impact on the image of the university, as well as expanding the model with new variables and limitations.
